# Classification of Signature-Based Phenotypes of Aging-Related Genes to Identify Prognostic and Immune Characteristics in HCC

**DOI:** 10.1155/2023/5735339

**Published:** 2023-03-20

**Authors:** Junjie Zhao, Chong Li, Qinggang Li, Shen Shen, Xiaobo Hu, Zihui Dong, Yize Zhang, Jiyuan Xing

**Affiliations:** ^1^Department of Pharmacy, The First Affiliated Hospital of Zhengzhou University, Zhengzhou, China; ^2^Department of Endocrinology and Metabolism, The First Affiliated Hospital of Zhengzhou University, Zhengzhou, China; ^3^Infectious Diseases Department, The First Affiliated Hospital of Zhengzhou University, Zhengzhou, China

## Abstract

Hepatocellular carcinoma (HCC), which has become one of the most significant malignancies causing cancer-related mortality, presents genetic and phenotypic heterogeneity that makes predicting prognosis challenging. Aging-related genes have been increasingly reported as significant risk factors for many kinds of malignancies, including HCC. In this study, we comprehensively dissected the features of transcriptional aging-relevant genes in HCC from multiple perspectives. We applied public databases and self-consistent clustering analysis to classify patients into C1, C2, and C3 clusters. The C1 cluster had the shortest overall survival time and advanced pathological features. Least absolute shrinkage and selection operator (LASSO) regression analysis was adopted to build the prognostic prediction model based on six aging-related genes (*HMMR*, *S100A9*, *SPP1*, *CYP2C9*, *CFHR3*, and *RAMP3*). These genes were differently expressed in HepG2 cell lines compared with LO2 cell lines measured by the mRNA expression level. The high-risk score group had significantly more immune checkpoint genes, higher tumor immune dysfunction and exclusion score, and stronger chemotherapy response. The results indicated that the age-related genes have a close correlation with HCC prognosis and immune characteristics. Overall, the model based on six aging-associated genes demonstrated great prognostic prediction ability.

## 1. Introduction

Hepatocellular carcinoma (HCC) has become one of the most common fatal malignancies in the world, accounting for nearly 90% of primary liver cancer cases [1]. Due to the lack of early clinical manifestations and complicated pathogenesis in HCC, it is usually diagnosed at an advanced stage with high metastatic rate [2]. Therefore, elucidating the molecular mechanism of HCC initiation and progression increases the potential to make a difference in improving the prognosis for HCC patients.

Aging is the principal factor for most chronic diseases, such as cancer, metabolic-associated diseases, or neurodegenerative diseases [3]. Therefore, targeting aging-associated signaling pathways can also provide clues to ameliorate aging-associated pathologies [4]. The existing studies have also demonstrated that cell senescence is associated with hyperproliferative conditions, including cancers, organ hypertrophy, fibrosis, and others [5]. The senescence-associated secretory phenotype involves multiple cell types and different hyperfunctions [5]. Cellular senescence, which is characterized by aging-related tissue dysfunction and multiple other conditions, represents a novel antitumor therapeutic approach. Previous studies identified that aging-related genes act as dominating risk factors in human cancers, associated with the initiation and progression of cancer patients [3]. Cellular senescence is a crucial regulatory mechanism, i.e., a double-edged sword involved in organismal aging and protecting against cancer at the same time [6].

Studies have reported that cellular senescence in the liver might enhance the clearance of hepatic stellate cells via innate and adaptive immunity activation [7]. To this end, aging-related genes and associated phenotypes have not been thoroughly explored; however, this is urgent for HCC patients to achieve individualized evaluation and provide effective treatment options.

## 2. Methods

### 2.1. Data Collection and Process

The HCC RNA-seq data were downloaded from the public database The Cancer Genome Atlas (TCGA) (https://www.cancer.gov/) with reference to Liver hepatocellular carcinoma (LIHC) via the TCGA GGJoy Dex Analysizer Application Programming Interface. In total, 365 samples from TCGA-LIHC were selected for our analysis, which was defined as the training set. GSE14520 and GSE76427 datasets were obtained from the Gene Expression Omnibus (GEO; https://www.ncbi.nlm.nih.gov/geo/info/datasets.html) and identified as validation datasets, with the independent selection of 221 and 115 patients, respectively. In addition, we adopted International Cancer Genome Consortium (ICGC)-LIRI-JP from the HCCDB database as the validation dataset.

### 2.2. Exploration of Aging-Related Genes

For this analysis, we downloaded the aging-associated genes from the CellAge (https://genomics.senescence.info/cells/) database, which included 279 human genes driving cellular senescence [8]. The CellAge is a database of genes associated with cell senescence.

### 2.3. Data Cleaning Process

All samples downloaded from public databases underwent a cleaning process by deleting incomplete clinical follow-up information, overall survival samples, and status samples. Data from the GEO database were matched with annotation information.

### 2.4. Classification of Aging-Associated Genes

The expression level of all aging-related genes was determined in LIHC samples. ConsensusClusterPlus was applied to divide these samples into different clusters based on the expression level of aging-associated genes [9]. In total, 500 bootstraps were conducted, and we set the number of clusters as 2–10.

### 2.5. Construction of Canadian Syncope Risk Score System

In order to construct the Canadian Syncope Risk (CSR) score system, we evaluated the Canadian Syncope Risk Score (CSRS) of each sample based on the formula CSRS = ∑*β*_*i*_ × Exp_*i*_, where *i* represents gene expression level, and *β* represents the Cox index. We classified our patients into high risk and low risk score subgroups [10].

### 2.6. Construction of Model for Aging-Associated Genes

In order to establish the risk score model based on aging-associated genes, we first recognized the differently expressed genes between different clusters. Second, the prognosis-related differently expressed genes were selected. The Least absolute shrinkage and selection operator (LASSO) Cox regression analysis was applied to identify the hub genes [11], and further dimension reduction analysis was performed to select the appropriate hub genes.

### 2.7. Immune Infiltration Evaluation

In order to estimate the immune cell infiltration level, we adopted tumor immune dysfunction and exclusion (TIDE) algorithms and evaluated the potential therapy response to immune checkpoint inhibitor therapy [12].

### 2.8. Gene Set Enrichment Analysis

Gene Set Enrichment Analysis (GSEA) analysis was utilized to explore the significantly different biological pathways between subtypes [13], and we adopted the candidate genes from the “Hallmark” database for further GSEA analysis.

### 2.9. Tumor Microenvironment Evaluation

Tumor microenvironment (TME) plays a vital role in the pathogenesis of various cancers, including HCC. In this study, we adopted the Cell-type Identification by Estimating Relative Subsets Of RNA Transcripts (CIBERSORT) algorithm (https://cibersort.stanford.edu/) to analyze the difference in immune infiltration and the immune microenvironment between the clusters [14]. This algorithm calculated the relative abundance of 22 immune cell types in each LIHC sample. ESTIMATE is a tool for predicting stromal score [15, 16], which refers to the presence of stroma in tumor tissue, with the immune score suggesting the infiltration of immune cells in tumor tissue and the estimated score indicating tumor purity. The CIBERSORT algorithms and ESTIMATE algorithm were adopted to clarify the cell composition of 22 immune cell types from HCC tissues based on genomic profiles.

### 2.10. Polymerase Chain Reaction Analysis

We collected the RNA of HepG2 and LO2 cell lines by RNeasy FFPE kit (QIAGEN, Hilden, Germany). The determination of nucleic acid concentration was carried out by NanoDrop 2000 (Thermo Fisher Scientific, Waltham, MA, USA). The RNA reverse transcription into complementary DNA (cDNA) was performed by HiScript III 1st Strand cDNA Synthesis Kit (+gDNA wiper; Vazyme, Nanjing, China). ChamQ Universal SYBR qPCR Master Mix (Vazyme) was employed for the quantitative real-time polymerase chain reaction (qRT-PCR) process. Glyceraldehyde-3-phosphate dehydrogenase (GAPDH) was treated as normal control sequence. The fluorescence quantitative PCR analysis was conducted by the Applied Biosystems RUO module (Applied Biosystems, San Francisco, CA, USA). The final data were analyzed with fold change =2^−△△CT^. The seven sequences were listed in Supplementary Table S1.

## 3. Results

### 3.1. Classification of LIHC Cohort Based on Aging-Associated Genes

**Figure 1:**
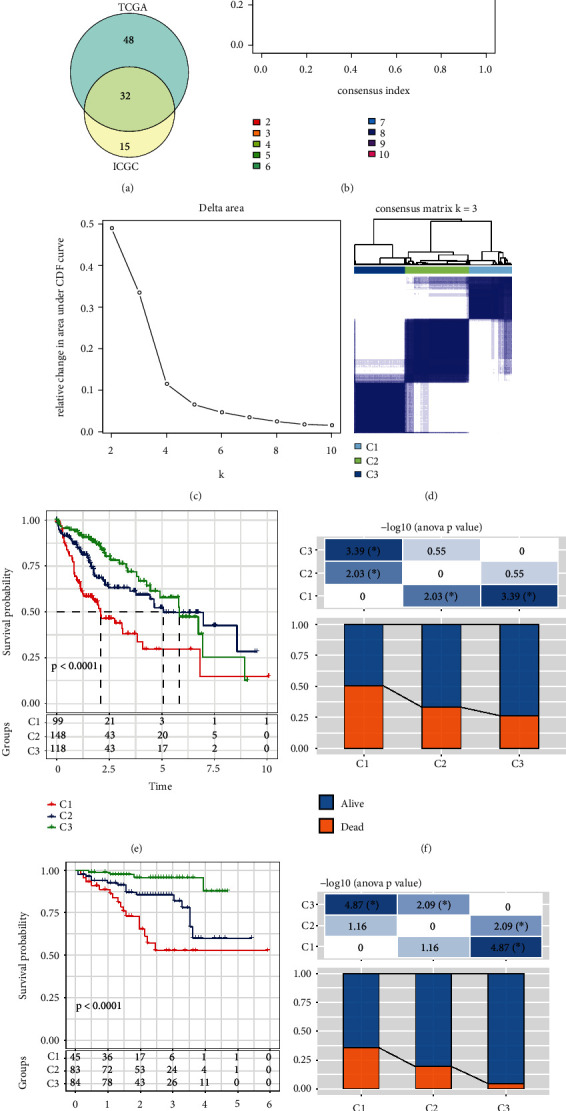
Self-consistent clustering analysis of aging-associated genes. (a) Overlap of differently expressed genes between the TCGA-LIHC and ICGC datasets. (b) CDF curve of the TCGA-LIHC samples, the CDF curve indicates the level of consensus and the stability of clustering. (c) CDF delta area curve of the TCGA-LIHC cohort. (d) Sample heatmap of self-consistent clustering analysis when *k* = 3. (e) Prognostic curve of three clusters. (f) Alive and dead information of three clusters. (g) Prognosis between three clusters in ICGC-LIHC. (h) Alive and dead status of three clusters in the ICGC LIHC cohort. ∗P < 0.05.

In order to explore the prognostic role of aging-associated genes in the LIHC cohort, we adopted gene expression profile spacing and Cox proportional hazard regression analysis and identified 80 HCC prognostic related genes in the TCGA database. In addition, we identified 47 prognostic related genes from the ICGC dataset. After comparison, there were 32 aging-associated genes in both datasets (Figure 1(a)). We further applied ConsensusClusterPlus analysis for 365 LIHC samples. The cumulative distribution function (CDF) delta area demonstrated that when “*k* = 3,” it reflected a more stable cluster model. We identified three clusters as cluster 1 (C1), cluster 2 (C2), and cluster 3 (C3; Figures 1(b), 1(c), and 1(d)). Furthermore, we investigated the prognosis between three clusters in TCGA-LIHC cohort and figured out that C1 cluster had shortest overall survival time compared with C2 and C3 (Figure 1(e)). The alive status percent in C1 was significantly decreased compared with C2 and C3 (Figure 1(f)). These results were also observed in ICGC cohort (Figures 1(g) and 1(h)).

### 3.2. Clinicopathological Features of the Three Clusters

In order to comprehensively distinguish the clinicopathological differences between the three clusters, we figured out that in the TCGA database, C1 compared with C3 had a higher percentage of advanced stages, and a lower proportion of early stages. Meanwhile, C1 had a higher advanced N stages proportion compared with early N stage, and a higher percentage of advanced M stages patients compared with C3. Tumor grades in C1 were notably different compared with C3. We also analyzed the viral etiology, and the results showed that C1 contained a higher percentage of Viral hepatitis type B (HBV) and Viral hepatitis type C (HCV) infected patients compared with C3. The proportion of patients younger than 60 years was significantly higher in C1 compared with C3 (Figure S1(a)). In the ICGC database, C1 contained more advanced stages compared with C2 and C3 (Figure S1(b)). These results demonstrated that the clinical signatures in C1 patients was significantly different compared with C3.

### 3.3. Mutation Signatures in the Three Clusters

**Figure 2:**
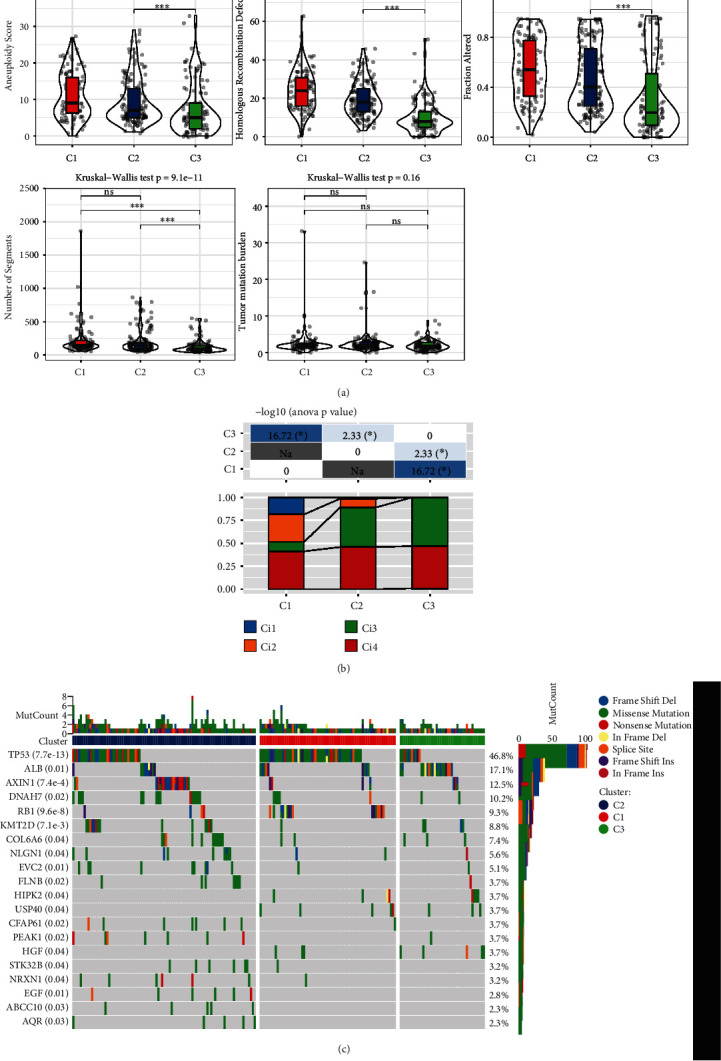
Genomic mutation profiles of three clusters. (a) Comparisons of the homologous recombination defects, aneuploidy score, altered fraction, number of segments, and tumor mutation burden in three clusters from TCGA. (b) Determination of immune molecular differences in three clusters. (c) Determination of cellular mutation differences in three clusters. ∗P < 0.05; ∗∗P < 0.01; ∗∗∗P < 0.001

First, we determined the molecular characteristics of TCGA-LIHC from pan-cancer analysis [17]. The analysis of mutation signatures demonstrated that C1 showed higher aneuploidy score (*p* = 1.5 × 10^−7^) homologous recombination defects (*p* = 5.5 × 10^−24^), altered fraction (*p* = 3.4 × 10^−11^), and number of segments (*p* = 9.1 × 10^−11^) compared with C3 (Figure 2(a)). We also compared the four clusters with immune signature-based four clusters, Ci1–Ci4 with the aging-related gene-based three clusters C1–C3, and the results showed that the aging-associated gene-based C3 obtained high frequency in the immune signature-based C3 cluster. The immune signature gene-based C3 cluster had better prognosis, whereas C4 and C6 had worse prognosis (Figure 2(b)). We also detected that genomic mutation and clusters had remarkably close correlations. The percent ranking of genes showing extensive mutations in HCC, from high to low, was TP53, ALB, AXIN, and DNAH3 (Figure 2(c)).

### 3.4. Immune Infiltration Features in the Three Clusters

**Figure 3:**
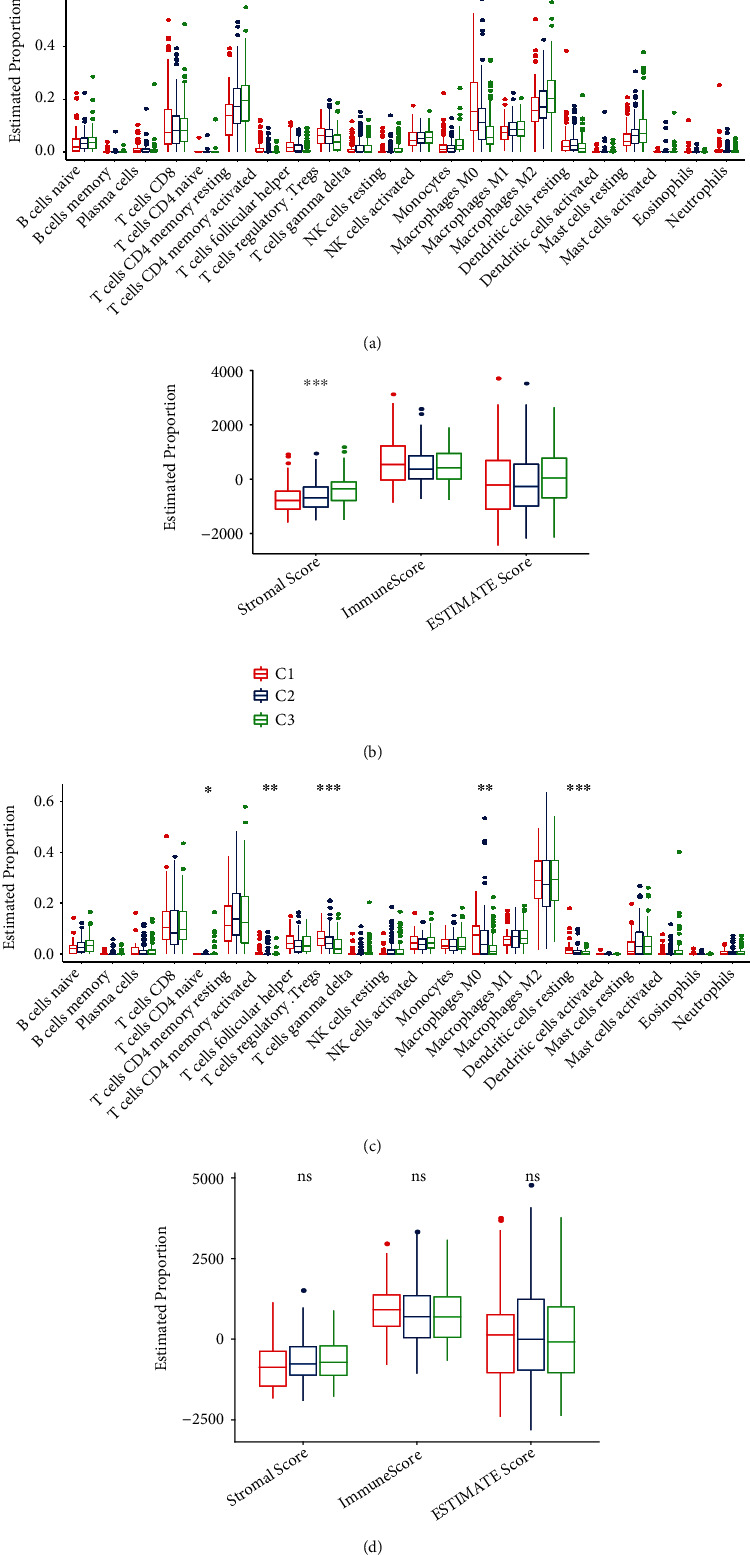
Evaluation of immune cell proportions for three clusters in two datasets. (a) Immune cell score difference between three clusters in the TCGA-LIHC cohort. (b) ESTIMATE immune infiltration difference between three clusters in the TCGA-LIHC cohort. (c) Immune cell score difference between three clusters in the ICGC-LIHC cohort. (d) ESTIMATE immune infiltration difference between three clusters in the ICGC-LIHC cohort. ∗P < 0.05; ∗∗P < 0.01; ∗∗∗P < 0.001.

In order to extensively describe the immune microenvironment differences in the three clusters, our analysis implicated that the relative abundance of 22 immune cells were remarkably different. Those of naïve B cells, memory B cells, CD4+ memory T resting and activated cells, T follicular helper, T regulatory, Natural killer (NK) cells-resting, monocytes, M0 macrophages, M2 macrophages, dendritic cells resting, mast cells, and eosinophils were considerably different (Figure 3(a)). We adopted ESTIMATE software to analyze the immune infiltrations in the three clusters and found that the stromal score was notably different between them (Figure 3(b)). Without exception, similar results were observed in the ICGC-LIHC cohorts. Naïve CD4+ T cells, memory-active CD4+ T cells, T cells regulatory-Treg, M0 macrophages, and resting dendritic cells showed significant differences between the three clusters. No significant difference was observed in stromal score, immune score, and ESTIMATE score (Figures 3(c) and 3(d)).

### 3.5. Enriched Signaling Pathways in the Three Clusters

In order to further investigate the signaling pathway differences between the clusters, we adopted the Hallmark database including recognized signaling pathways [18]. The GSEA analysis indicated that C1 versus C3 was significantly enriched in 12 signaling pathways, such as MYC-TARGETS V1, MITOTIC SPINDLE, E2F TARGETS, G2M CHECKPOINT, UNFOLDED PROTEIN RESPONSE, DNA REPAIR, MYC TARGETS V2, PROTEIN SECRETION, PI3K AKT MTOR SIGNALING, ALLOGRATE REJECTION, MTORC SIGNALING, and SPERMATOGENESIS in the TCGA database (Figure S2(a)). In ICGC-LIHC, there were 19 signaling pathways that were significantly enriched between C1 and C3 (Figure S2(b)). We also compared C1 versus C2 and C2 versus C3 in the TCGA-LIHC cohort (Figures S2(c) and S2(d)). The result suggested that C1 was enriched in cell cycle-associated pathways, and that cell aging-associated genes might play important roles in the cell cycle, which might also strongly influence the TME.

### 3.6. Identification of Aging-Associated Hub Genes

**Figure 4:**
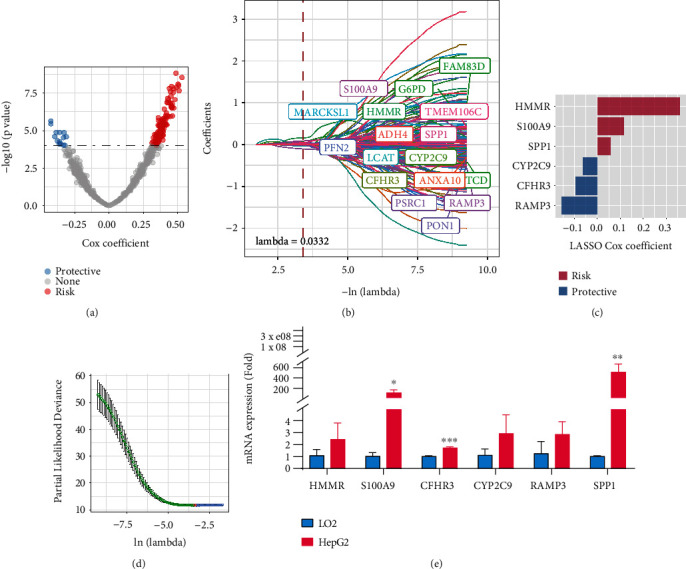
Construction of aging-associated gene-based prognostic model. (a) Differently expressed genes among the three clusters. In total, 139 genes were selected. The red represents risk factor genes, and the blue represents protective roles. (b) The curve of coefficients changes with the value of lambda. (c) The partial likelihood deviance varies with the value of lambda. (d) Distribution of LASSO coefficients of the senescence-related gene signature. (e) mRNA expression of six senescence-related genes. ∗P < 0.05; ∗∗P < 0.01; ∗∗∗P < 0.001.

Next, we applied the “limma” R package to calculate the differently expressed genes, with FDR <0.05 and log2FC >1, to finally select 611 genes. Using univariate regression analysis, we found 137 prognosis-related genes (*p* < 0.001) including 125 risk genes and 14 protective genes (Figure 4(a)). Furthermore, we adopted LASSO regression analysis to select the best fitting hub genes. The trajectories of independent variables were shown in Figure 4(b). With the increase of lambda, the independent coefficient also gradually rises. Ten-fold cross validation was applied to construct the gene model, and the confidence interval under each lambda was calculated (Figure 4(c)). The figure illustrates that when lambda = 0.0332, the model reaches the optimal value. A total of 17 genes with lambda = 0.0332 were selected as the target genes for the next step. Multivariate analysis was performed by stepwise logistic regression analysis. Finally, we identified six genes, including *HMMR*, *S100A9*, *SPP1*, *CYP2C9*, *CFHR3*, and *RAMP3* as prognosis associated genes based on aging signature-associated classifications (Figure 4(d)). Furthermore, we validated the expression of these genes by qPCR analysis, and the results indicated that S100A9, CFHR3, and CYP2C9 were significantly highly expressed in HepG2 cell lines compared with LO2 cell lines (Figure 4(e)).

### 3.7. Construction and Validation of Risk Score Prognosis Model Based on Cellular Senescence-Related Signatures

**Figure 5:**
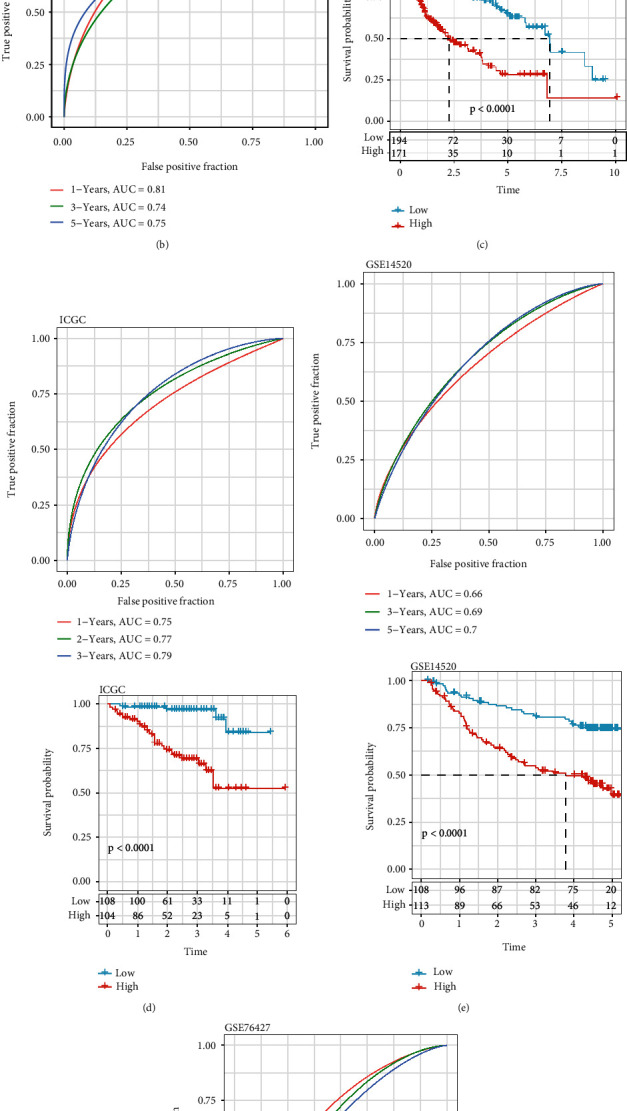
CSRS estimation, validation, and prognostic prediction analysis. (a) CSRS, overall survival time, alive status, and senescence-associated genes. (b) CSRS-based ROC curve and AUC curve comparisons in the TCGA-LIHC cohort. (c) CSRS-based ROC curve and AUC curve comparisons in the TCGA-LIHC cohort. (d) Overall survival time of high and low risk score groups in the ICGC-LIHC cohort. (e) ROC curve and overall survival time of high and low risk score groups in the GSE14520 cohort. (f) ROC curve and overall survival time of high and low risk score groups in the GSE6427 cohort.

We calculated the CSRS of each sample and normalized these values, and the high-risk score patients obtained higher mortality status (Figure 5(a)). We applied Receiver operating characteristic curve (ROC) analysis on the CSRS -based classification, and the prognostic prediction classification efficiencies for 1, 3, and 5 years, respectively, were illustrated in Figure 5(b). This CSRS-based classification had a high Area under ROC curve (AUC) line area, indicating that this model had good prediction efficacy.

We further analyzed the overall survival time between high CSRS group and low CSRS group. The survival curve suggested that the high CSRS group had shorter overall survival time compared with the low CSRS group (Figure 5(c)). To test the stability of this CSRS-based prognostic model, we applied the same method on the ICGC-LIHC cohort. The high CSRS group had poor prognosis (Figures 5(d), 5(e), and 5(f)).

### 3.8. Clinicopathological Features between the High and Low CSRS Groups

We calculated the CSRS of each sample and classified them by T stage, N stage, M stage, clinical stage, grade, viral etiology, fibrosis age, gender, status, and primary clusters. The results demonstrated that advanced T stages, higher grades, and clinical stages had higher CSRSs. In addition, patients with virus infection had higher CSRS. The C1 cluster had significantly higher CSRSs compared with C2 and C3 (Figure S3(a)). We also compared the prognosis difference between the various clinicopathological features as defined by CSRS. The results suggested that the CSRS-based classification had good stability and efficiency (Figure S3(b)).

### 3.9. Immune Infiltration and Enriched Signal Pathways

**Figure 6:**
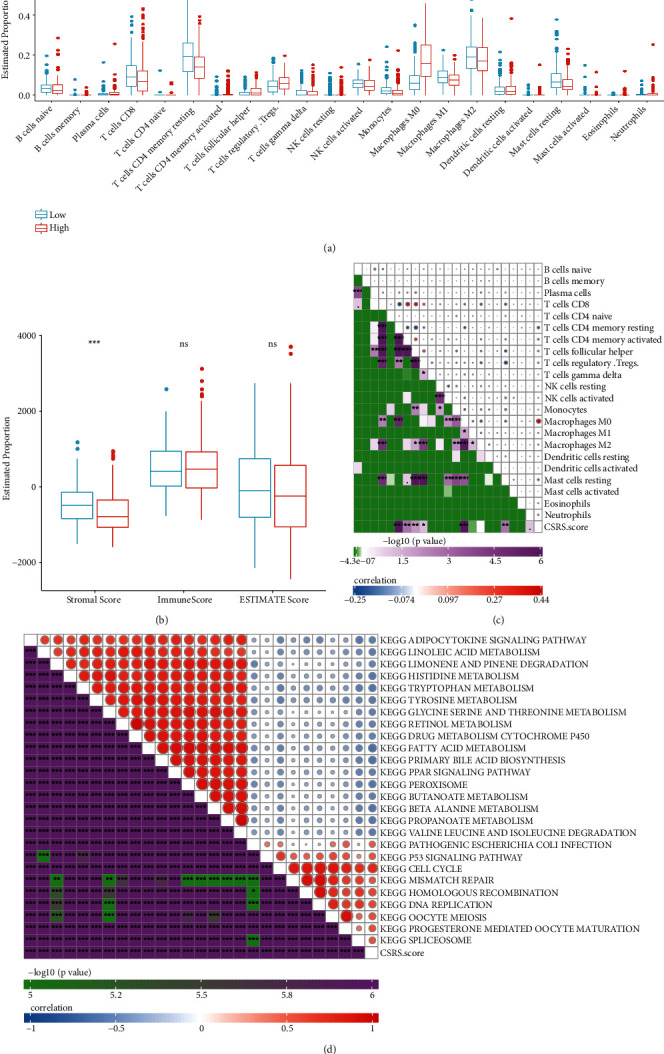
Immune and signaling pathways featured in high and low CSCR score subgroups. (a) Immune cells components and their proportion between high and low subgroups in the TCGA LIHC cohort. (b) Correlation analysis of proportion and CSCR score of 22 immune cells. (c) The CSRS and immune cells had significantly close relationship. (d) The low CSRS subgroup was profoundly enriched in the P53 signal pathway, cell cycle, mismatch repair, homologous recombination, and DNA replication. ∗P < 0.05; ∗∗P < 0.01; ∗∗∗P < 0.001.

In order to clarify the differences in the immune microenvironment within the CSRS-based classification, we compared the relative abundance of 22 immune cell types (Figure 6(a)). The results indicated that the infiltration level of immune cells was significantly different between high and low CSRS subgroups except for CD4+ T cells, T gamma-delta cells, resting NK cells, M2 macrophages, resting dendritic cells, activated dendritic cells, and activated mast cells (Figure 6(a)). The ESTIMATE analysis revealed that the high CSRS group had significant lower estimated proportions in stromal score (Figure 6(b)). The CSRS and immune cells had significantly close relationship (Figure 6(c)). The GSEA analysis supported the notion that, in the classification based on high and low CSRS, significantly differently expressed genes were profoundly enriched in the P53 signal pathway, cell cycle, mismatch repair, homologous recombination, and DNA replication (Figure 6(d)).

### 3.10. Differences in Immune Therapy and Chemical Therapy between the High and Low CSRS Subgroups

The expression of immune checkpoint genes was significantly different between the two subgroups (Figure S4(a)). Overall, most of the immune checkpoint genes were highly expressed in the high CSRS group. We calculated the immune therapy responses between the high and low CSRS groups, and the results revealed that the high CSRS subgroup had higher TIDE score, suggesting that this score group had higher probability of immune escape and lower possibility of immunotherapy benefits (Figure S4(b)). In addition, we observed that the high CSRS cohort was more sensitive to docetaxel, cisplatin, cytarabine, and bortezomib chemical therapies (Figure S4(c)).

### 3.11. Adjustment of the CSRS-Based Prognostic Model

**Figure 7:**
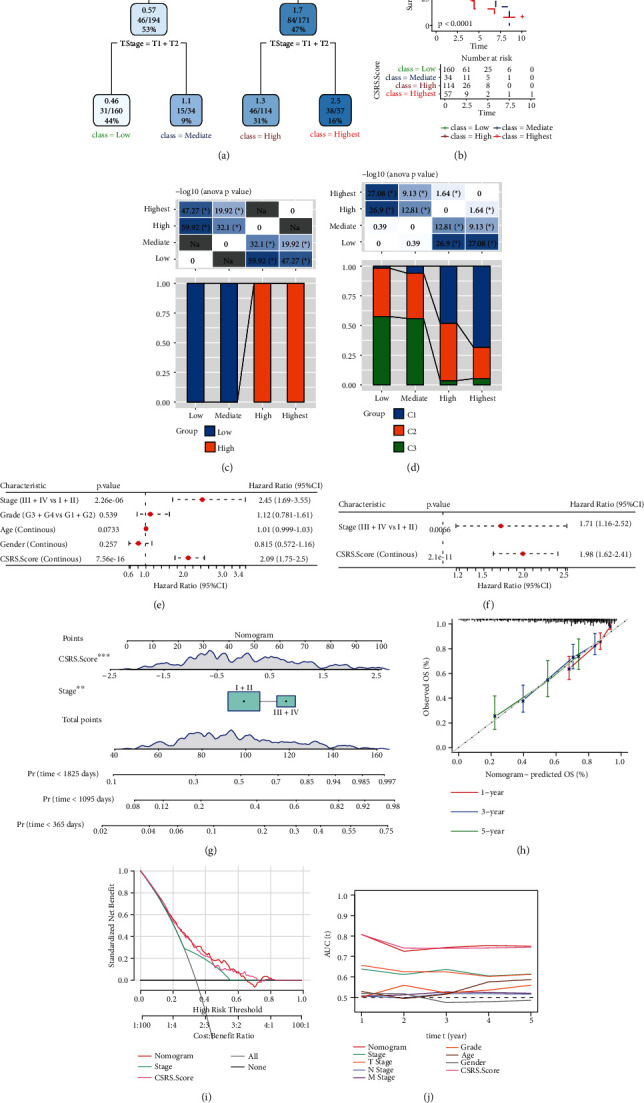
Risk-score model adjustment based on clinical features. (a) The decision tree was constructed according to age, sex, disease stage, T stage, grade, and CSRS. Score of HCC patients in the TCGA-LIHC cohort. (b) Overall survival time curve between subgroups based on decision tree (low, mediate, high, and highest). (c) Comparisons of CSCR score-based risk score subgroup and decision tree-based subgroup classification (low, mediate, high, and highest). (d) Comparisons of differently expressed genes identified in subgroups and decision tree classification (low, mediate, high, and highest). (e and f) Univariate and multivariate Cox analyses of CSRS and clinicopathological features. (g) Line diagram model. (h) Calibration curves for 1, 3, and 5 years of the row diagram. (i) Decision curve of the line graph. (j) Comparison with other clinicopathological features. ∗P < 0.05; ∗∗P < 0.01; ∗∗∗P < 0.001.

We constructed a decision tree based on age, sex, stage, T stage, grade, and CSRS in the TCGA-LIHC cohort. This analysis demonstrated that only the CSRS and T stage were left in the decision tree, which classified samples into four risk score subgroups (Figure 7(a)). There were significant differences in overall survival between these risk subgroups (Figure 7(b)). We also discovered significant differences in our novel subtypes. The highest subgroup contained a higher percentage of C1 subtypes (Figures 7(c) and 7(d)). The univariate and multivariate Cox regression analyses of CSRS and clinicopathological features showed that CSRS was the most significant prognostic factor (Figures 7(e) and 7(f)). To quantify the risk assessment and survival probability of HCC patients, we adopted the CSRS with other clinicopathological features to establish a nomogram (Figure 7(g)). This result suggested that the CSRS had the greatest impact on survival prediction. Furthermore, we adopted the calibration curve to evaluate the accuracy of this model (Figure 7(h)). The 1-, 3-, and 5-year results implied that this nomogram had good efficiency in prognostic performance. The decision curve uncovered that both the CSRS and the nomogram showed the strongest survival prediction ability (Figures 7(i) and 7(j)).

## 4. Discussion

Aging has been widely recognized as a significant risk factor for cancers. Cellular senescence plays an important role in the aging process and in cancer initiation and progression. However, the correlations between cancer and the aging microenvironment have not been fully explored. Here, we described the expression profiles of aging-associated genes in HCC. Based on these genes, we applied self-consistent clustering analysis and identified three clusters and their corresponding prognostic features, pathological features, and immune signatures. In addition, we performed LASSO analysis and recognized six aging-associated hub genes, namely *HMMR*, *S100A9*, *SPP1*, *CYP2C9*, *CFHR3*, and *RAMP3*, which contributed to building an effective prognostic model.

The aging process is associated with low-grade inflammation [19]. From the cellular perspective, senescence is closely linked to inflammation, and the immune and cancer microenvironment [20]. Adaptive immune cells such as monocytes, neutrophils, macrophages, and NK cells exhibit significant phenotypic modulation and relative frequency during the aging process. Recent studies have demonstrated that aging might affect immune cells' mitochondrial activities, inducing inflammation, and impairing their intracellular bacteria-killing capability [21]. In the present analysis, we identified three aging-associated clusters, and the aging gene-based C1 cluster exhibited the shortest overall survival time, highest immune score, and maximum immune infiltration compared with other subtypes. Li and co-workers figured out that an aging-related model had a good risk prediction efficiency in rectal cancer prognosis [22]. However, the links between aging-associated changes and cancer initiation and progression have not been fully explored [23]. Some ageing processes might accelerate cancer initiation and development processes, whereas others might inhibit tumor progression [23]. Studies have demonstrated that tumor formation might combine with tumor formation through DNA damage response [24–26], telomeres, replicative senescence and tissue homeostasis, endocrine changes, immune system aging, vascular ageing, and angiogenesis [23]. In our study, the prognostic model based on aging-associated genes suggested that the high CSRS group had poor prognosis. This score group also presented higher TIDE score, which indicated the high probability of escape and low benefit from immunotherapies for this group. Similarly, in a triple-negative breast cancer analysis, a risk model of a high-risk score group based on 10 aging-related genes presented poor prognosis compared with a low risk score group [27]. In contrast to our results, Zhai et al. figured out that, in lung squamous carcinoma, the high-risk group based on aging-related genes benefit from immunotherapy compared with the low risk score group [28]. Chen et al. also analyzed the aging-related genes in HCC. They selected seven aging-related genes POLA1, CDK1, SOCS2, HDAC1, MAPT, RAE1, and EEF1E1 by LASSO analysis. They also build a risk score model, which had good efficiency in prognosis prediction. In our model we also adopted aging-associated genes, we first classified HCC patients into three clusters based on aging-associated gene transcription. Furthermore, we also adopted the LASSO regression analysis and selected six aging-related genes (*HMMR*, *S100A9*, *SPP1*, *CYP2C9*, *CFHR3*, and *RAMP3*). These aging-related gene associated risk score model had good efficiency in prognosis prediction and immune characteristics [29].

Certain limitations of this analysis remain to be addressed. We built this aging-associated gene-based prognostic model using public databases and bioinformatics analysis. Thus, the results still need further validation and intensive exploration. In addition, the relationship between aging signatures and immune infiltration, immune response, and pathological features also require stronger clinical data support. Finally, the mechanism between aging, immune microenvironment, and cancer still need further exploration and validation.

## 5. Conclusion

Collectively, aging signatures play important roles in HCC progression and prognosis. The prognostic model based on HMMR, S100A9, SPP1, CYP2C9, CFHR3, and RAMP3 had efficient prediction ability, which might provide individual therapeutic recommendations for HCC.

## Data Availability

Data supporting this research article are available from the corresponding author or first author on reasonable request.

## Supplementary Materials

Supplementary Materials Figure S1 Evaluation of clinical pathology features in three clusters. (a) Comparison of clinical pathology features between three clusters in the TCGA-LIHC cohort. (b) Comparison of clinical pathology features between three clusters in the ICGC-LIHC cohort. Figure S2 GSEA analysis in three clusters. (a) GSEA analysis results for C1 versus C3 in the TCGA-LIHC cohort. (b) Bar plot of GSEA analysis for C1 versus C3 in the TCGA-LIHC cohort. (c) Plot illustrating the GSEA analysis of enriched signal pathways in the three clusters. (d) GSEA analysis of C1 versus C2 and C2 versus C3; the enriched signal pathways were illustrated in this plot in the TCGA cohort. (e) GSEA analysis of C1 versus C2 and C2 versus C3; the enriched signal pathways were illustrated in this plot in the ICGC cohort. Figure S3 CSCR score evaluation based on different clinical features. (a) The high and low CSCR score subgroups had significant different clinical pathologies. (b) Overall survival time between high and low risk score subgroups based on different clinical pathological classification. Figure S4 Difference in immunotherapies and chemical therapies between the high and low risk score subgroups. (a) Immune check point genes' expression profiles of high and low risk score subgroups in the TCGA-LIHC cohort. (b) TIDE estimation between the high and low risk score groups. (c) Estimated IC50 of pocetaxel, paclitaxel, cisplatin, cytarabine, bortezomib, and gefitinib between the high and low CSCR score groups in the TCGA-LIHC cohort. Table S1. The information of primers sequences for qRT-PCR assay.
