# Supplementary material for: Classification of Signature-Based Phenotypes of Aging-Related Genes to Identify Prognostic and Immune Characteristics in HCC
Source: Anal Cell Pathol (Amst). 2023 Mar 20;2023:5735339. doi: 10.1155/2023/5735339 (PMC10042640; doi:10.1155/2023/5735339)
Supplement: Supplementary Materials — Figure S1 Evaluation of clinical pathology features in three clusters. (a) Comparison of clinical pathology features between three clusters in the TCGA-LIHC cohort. (b) Comparison of clinical pathology features between three clusters in the ICGC-LIHC cohort. Figure S2 GSEA analysis in three clusters. (a) GSEA analysis results for C1 versus C3 in the TCGA-LIHC cohort. (b) Bar plot of GSEA analysis for C1 versus C3 in the TCGA-LIHC cohort. (c) Plot illustrating the GSEA analysis of enriched signal pathways in the three clusters. (d) GSEA analysis of C1 versus C2 and C2 versus C3; the enriched signal pathways were illustrated in this plot in the TCGA cohort. (e) GSEA analysis of C1 versus C2 and C2 versus C3; the enriched signal pathways were illustrated in this plot in the ICGC cohort. Figure S3 CSCR score evaluation based on different clinical features. (a) The high and low CSCR score subgroups had significant different clinical pathologies. (b) Overall survival time between high and low risk score subgroups based on different clinical pathological classification. Figure S4 Difference in immunotherapies and chemical therapies between the high and low risk score subgroups. (a) Immune check point genes' expression profiles of high and low risk score subgroups in the TCGA-LIHC cohort. (b) TIDE estimation between the high and low risk score groups. (c) Estimated IC50 of pocetaxel, paclitaxel, cisplatin, cytarabine, bortezomib, and gefitinib between the high and low CSCR score groups in the TCGA-LIHC cohort. Table S1. The information of primers sequences for qRT-PCR assay. [file 5735339.f1.doc]

**Supplementary Figure 1**

**
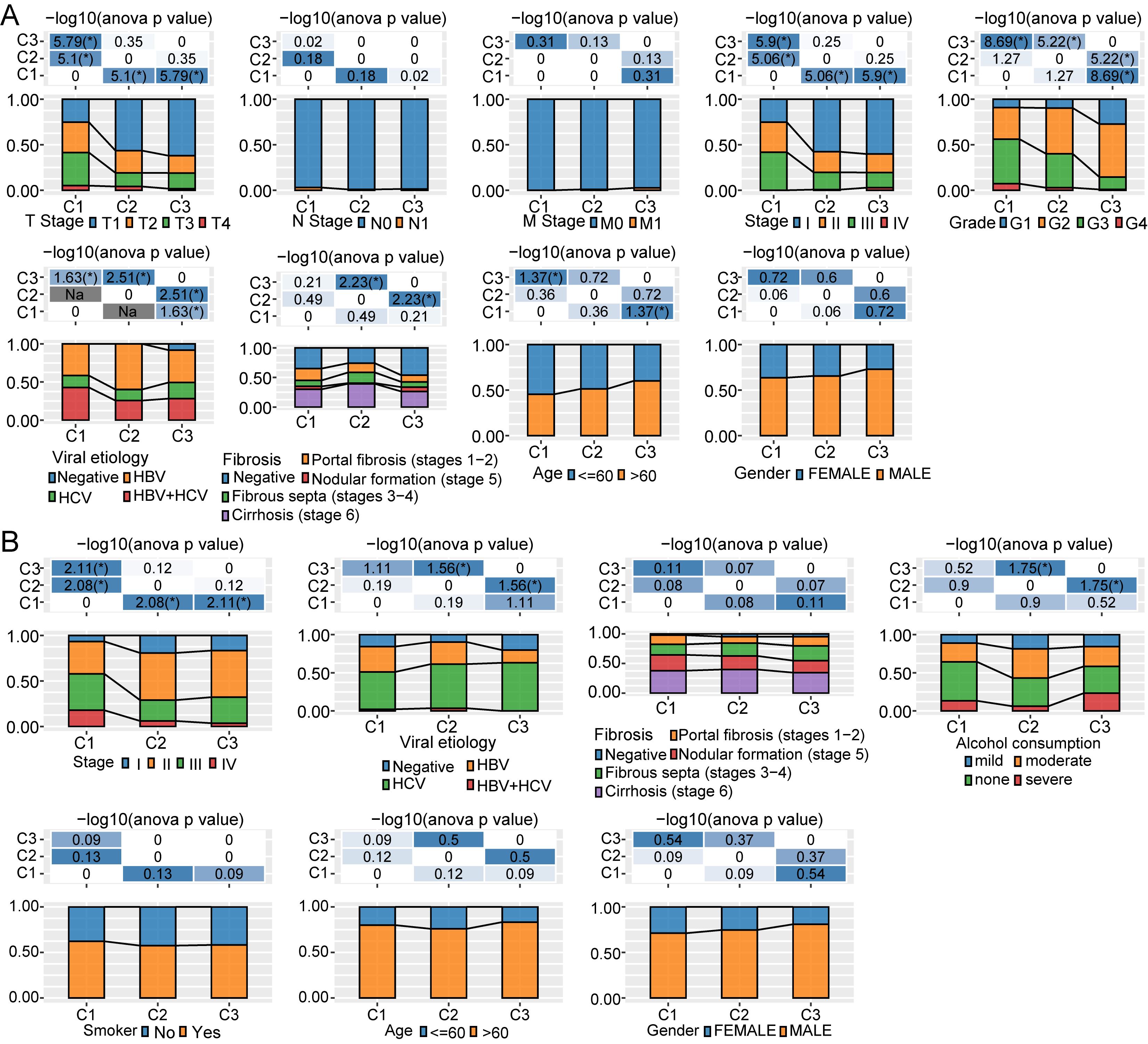
**

**Supplementary Figure 1 Evaluation of clinical pathology features in 3 clusters**

A. Comparison of clinical pathology features between 3 clusters in the TCGA-LIHC cohort. B. Comparison of clinical pathology features between 3 clusters in the ICGC-LIHC cohort.

**Supplementary Figure 2**


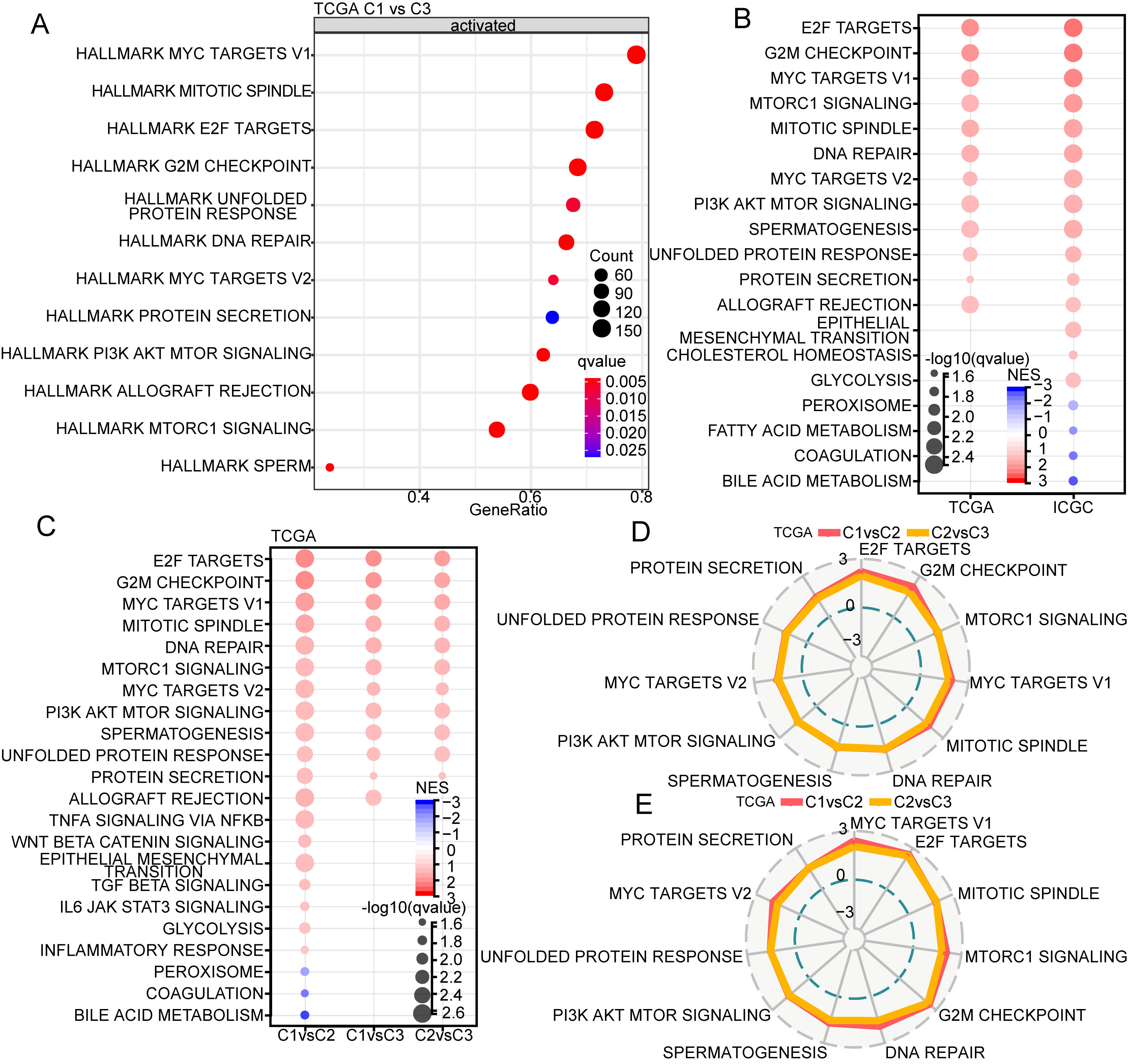


**Supplementary Figure 2 GSEA analysis in 3 clusters**

A. GSEA analysis results for C1 vs. C3 in the TCGA-LIHC cohort. B. Bar plot of GSEA analysis for C1 vs. C3 in the TCGA-LIHC cohort. C. Plot illustrating the GSEA analysis of enriched signal pathways in the 3 clusters. D. GSEA analysis of C1 vs. C2, C2 vs. C3; the enriched signal pathways were illustrated in this plot in the TCGA cohort. E. GSEA analysis of C1 vs. C2, C2 vs. C3; the enriched signal pathways were illustrated in this plot in the ICGC cohort.

**Supplementary Figure 3**

**
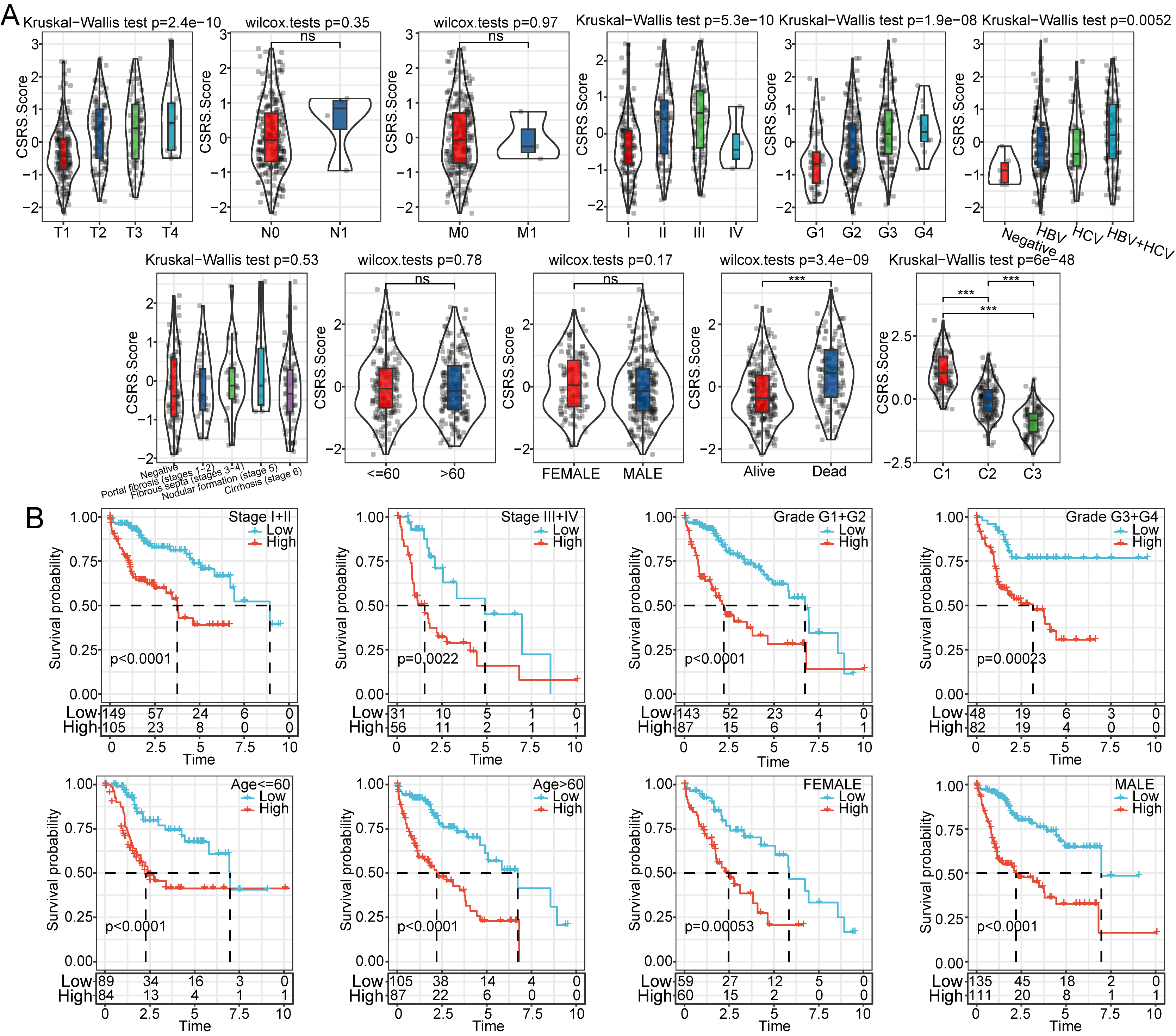
**

**Supplementary Figure 3 CSCR score evaluation based on different clinical features**

A. The high and low CSCR score subgroups had significant different clinical pathologies. B. Overall survival time between high and low risk score subgroups based on different clinical pathological classification.

**Supplementary Figure 4**

**
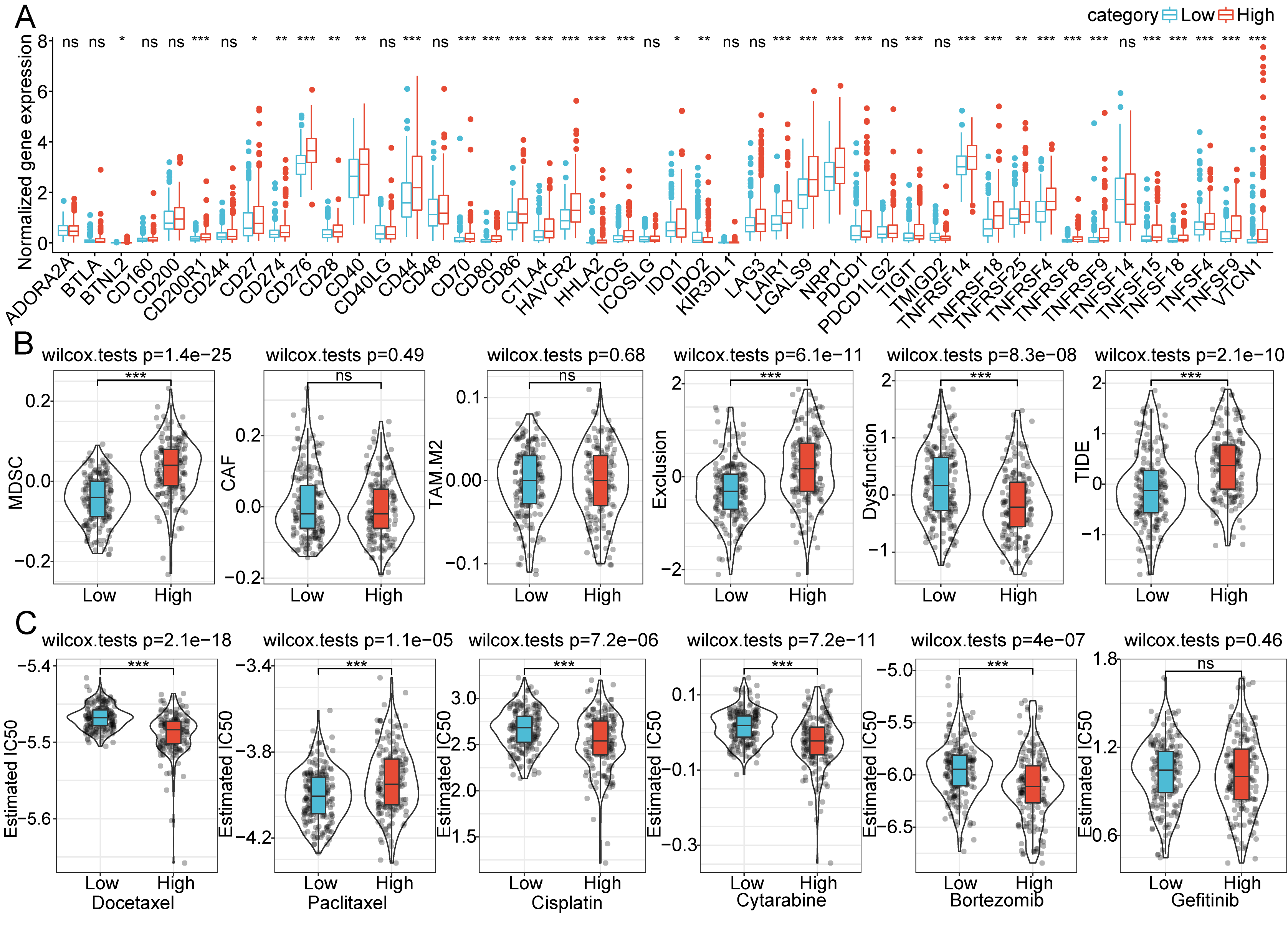
**

**Supplementary Figure 4 Difference in immunotherapies and chemical therapies between the high and low risk score subgroups**

A. Immune check point genes’ expression profiles of high and low risk score subgroups in the TCGA-LIHC cohort. B. TIDE estimation between the high and low risk score groups. C. Estimated IC50 of pocetaxel, paclitaxel, cisplatin, cytarabine, bortezomib, and gefitinib between the high and low CSCR score groups in the TCGA-LIHC cohort.

**Supplementary Table 1. The information of primers sequences for qRT-PCR assay.**

| **Primer name** | **Sequence (5'-3')** |
| --- | --- |
| GAPDH-R | GTGTCGCTGTTGAAGTCAGAGGAG |
| GAPDH-F | CAAGGCTGTGGGCAAGGTCATC |
| HMMR-R | GGCTATTTTCCCTTGAGACTCT |
| HMMR-F | AAGAGAAACAAAGATGAGGGGT |
| S100A9-R | TGCTTGTCTGCATTTGTGTCC |
| S100A9-F | TCAAAGAGCTGGTGCGAAAA |
| CFHR3-R | GGCATTGGTACTCGACTCTTGACTG |
| CFHR3-F | CTCTACAGAAGTTGCCTGCCATCC |
| CYP2C9-R | GGGCTTCCTTCACTGCTTCATATCC |
| CYP2C9-F | CTTGTGGTCCTTGTGCTCTGTCTC |
| RAMP3-R | GACAGTCAGAACGACGGGTATAACG |
| RAMP3-F | TTTCGCAGACATGATGGGCAAGG |
| SPP1-R | CAGGTCTGCGAAACTTCTTAGAT |
| SPP1-F | CTCCATTGACTCGAACGACTC |
